# Association of dietary *n* - 3 polyunsaturated fatty acids with breast cancer risk: Serial mediating roles of erythrocyte *n* - 3 polyunsaturated fatty acids

**DOI:** 10.3389/fnut.2022.990755

**Published:** 2022-11-03

**Authors:** Zhuolin Zhang, Yiling Jiang, Xue Li, Dandan Shi, Ting Ma, Ruolin Zhou, Caixia Zhang

**Affiliations:** ^1^Department of Epidemiology, School of Public Health, Sun Yat-sen University, Guangzhou, China; ^2^Guangdong Provincial Key Laboratory of Food, Nutrition and Health, School of Public Health, Sun Yat-sen University, Guangzhou, China

**Keywords:** *n* **–** 3 polyunsaturated fatty acids, breast cancer, diet, erythrocyte, mediation analysis

## Abstract

**Background:**

Dietary *n* **–** 3 polyunsaturated fatty acids (PUFAs) were found to be inversely associated with breast cancer risk; however, the underlying pathways between them remain uncertain. We aimed to explore serial mediatory roles of erythrocyte *n* **–** 3 PUFAs in association between dietary *n* **–** 3 PUFAs and breast cancer risk.

**Materials and methods:**

Using a case-control study, 850 cases and 861 controls completed structured questionnaires with dietary information. Erythrocyte *n* **–** 3 PUFAs were measured by gas chromatography. Odds ratios (ORs) and 95% confidence intervals (CIs) were obtained using multiple unconditional logistic regression models to examine association between dietary *n* **–** 3 PUFAs and breast cancer risk. Mediation analyses with bootstrapping were conducted to investigate indirect effects.

**Results:**

Higher intake of dietary ALA, long-chain *n* **–** 3 PUFAs and total *n* **–** 3 PUFAs was associated with lower risk of breast cancer. The adjusted OR*_*tertile 3 v.1*_* (95% CI) was 0.70 (0.55, 0.90) for ALA, 0.76 (0.60, 0.97) for long-chain *n* **–** 3 PUFAs and 0.74 (0.58, 0.94) for total *n* **–** 3 PUFAs, respectively. Mediation analysis showed that erythrocyte long-chain *n* **–** 3 PUFAs served as sequential mediators in the relationship between dietary long-chain or total *n* **–** 3 PUFAs and breast cancer risk. In particular, erythrocyte long-chain *n* **–** 3 PUFAs completely mediated the association between dietary long-chain *n* **–** 3 PUFAs and breast cancer risk [indirect effect (95% CI) = –0.982 (–1.529, –0.508)]. The relationship between dietary total *n* **–** 3 PUFAs and breast cancer risk was partly mediated by erythrocyte long-chain *n* **–** 3 PUFAs [indirect effect (95% CI) = –0.107 (–0.216, –0.014)], accounting for 19.31%. However, the serial mediation model in dietary ALA and risk of breast cancer was not statistically significant [indirect effect (95% CI) = –0.042 (–0.144, 0.049)].

**Conclusion:**

This study highlights the complexity and inaccuracy in using a simple analysis of individual dietary *n* **–** 3 PUFAs to examine their associations with breast cancer risk without considering the variety of metabolic processes. Interventions aimed at increasing erythrocyte long-chain *n* **–** 3 PUFAs may represent a promising strategy for breast cancer prevention.

## Introduction

Breast cancer is the most commonly diagnosed cancer and the fifth leading cause of cancer mortality globally in women worldwide (1). It is also the most common cancer among Chinese women, with an estimated 429,105 new cases in 2022 (2). Data on the association of various dietary nutrients, including *n* **–** 3 polyunsaturated fatty acids (*n* **–** 3 PUFAs), with the occurrence of breast cancer have long been increasing (3). Women with a higher dietary *n* **–** 3 PUFA intake are at a decreased risk of breast cancer (4, 5). However, the potential protective mechanisms of dietary *n* **–** 3 PUFAs on breast cancer risk are not clear.

Emerging evidence has revealed that some types of dietary *n* **–** 3 PUFAs are positively associated with circulating *n* **–** 3 PUFAs (6, 7). However, factors such as genetic variations, senility, blood lipids, alcohol consumption might also influence the levels of circulating *n* **–** 3 PUFAs (8). Moreover, the correlation between dietary α-linolenic acid (ALA) and ALA in circulating blood was relative low (9). Plasma docosapentaenoic acid (DPA) was not found to be significantly related to dietary DPA (10). Although an increased consumption of *n* **–** 3 PUFAs is generally associated with an increase of *n* **–** 3 PUFAs composition of blood, this remains unsure for ALA and DPA.

There is a series of conversion process of *n* **–** 3 PUFAs in the body. ALA is endogenously elongated and desaturated into eicosapentaenoic acid (EPA) by delta-6 and delta-5 desaturase and elongase-5 enzymes (11). Next, EPA is elongated to docosapentaenoic acid (DPA), which is then desaturated and finally produces docosahexaenoic acid (DHA) through the Sprecher pathway. Moreover, the delta-4 desaturation can also produce DHA from EPA in mammalian and human cells (12). It was reported that higher consumption of ALA might increase the rate of ALA oxidation, limit its accumulation in circulation and reduce the rate of conversion to EPA (13). Additionally, studies showed that dietary long-chain *n* **–** 3 PUFAs could down-regulate the conversion of plasma ALA to long-chain *n* – 3 PUFAs (13, 14). Furthermore, the conversion from ALA to DHA could be increased by the absence of dietary DHA (15). Therefore, the possible mechanism of the relationship between dietary *n* **–** 3 PUFAs and breast cancer risk might involve the conversion of ALA to EPA, DPA, and/or DHA in the body (13); as well as the influence of dietary *n* **–** 3 PUFAs on the conversion process of circulating *n* **–** 3 PUFAs.

Human erythrocytes have a life-span in the circulation of approximately 120 days, which can reflect dietary intake over several months and represent an integrative measure of the interaction concerning dietary, metabolic, and genetic factors (16). Therefore, erythrocyte *n* **–** 3 PUFAs were usually regarded as biomarkers of dietary *n* **–** 3 PUFAs. Mediation analysis investigates the mechanisms of the observed relationships between the independent and dependent variables and examines how they relate to the mediating variables (17). In terms of serial mediation, it has been hypothesized that variables affect each other sequentially. Such analysis will enable us to estimate the potential mediating roles of erythrocyte *n* **–** 3 PUFAs. It will also contribute to examine whether each erythrocyte *n* **–** 3 PUFA play an independent role in breast cancer risk or play roles after being converted to other types of *n* **–** 3 PUFAs via metabolic enzymes.

In this context, this study aimed to investigate whether erythrocyte *n* **–** 3 PUFAs would be sequential mediators in the association between dietary *n* **–** 3 PUFAs and breast cancer risk. The results of the mediation analysis may help to provide insight into the mechanisms underlying the protective effect of dietary *n* **–** 3 PUFAs on breast cancer risk and provide research basis for developing targeted cancer prevention strategies. This will cultivate the interest in erythrocyte *n* **–** 3 PUFAs as comprehensive biomarkers related to breast cancer and provide dietary nutritional modification strategies for breast cancer patients.

## Materials and methods

### Study population

Detailed description for this hospital-based case-control study have been published elsewhere (18). Briefly, eligible breast cases were recruited from two hospitals between September 2011 and December 2019. The inclusion criteria for cases were as follows: females aged 25–70 years with newly diagnosed and histologically confirmed breast cancer no more than 3 months before the interview, natives in Guangdong or having lived in Guangdong for at least 5 years, and understanding or speaking Mandarin/Cantonese. In total, 1,677 of 1,884 eligible cases were successfully interviewed (89.01% response rate). Among them, 869 participants provided blood samples and 853 blood samples were adequate for laboratory analyses. Participants with missing information on other covariates were excluded and leave 850 breast cancer cases were included in the final analysis.

Control subjects were female patients without breast cancers and were simultaneously recruited from the same hospitals as the cases. Patients were excluded if they had a prior history of any cancer or did not understand or speak Mandarin/Cantonese. Totally, 1,762 of 1,965 control subjects were recruited with a response rate of 89.67%. Eight hundred and ninety-two controls provided sufficient blood samples for fatty acid measurement. Finally, 861 control subjects, frequency-matched to cases by 5-year age intervals, were included in the analysis.

### Data collection

Information on demographic characteristics, anthropometry factors, lifestyle behaviors, first-degree relatives with cancer, menstrual and reproductive history were collected by trained interviewers through face-to-face interviews. Regular smoking was defined as smoking at least one cigarette/day for more than 6 months. Passive smoking was defined as exposure to the smoke from smokers for at least 15 mins/day in the past 5 years. Regular drinking was defined as drinking alcohol at least once a week over the past year. Menopausal status was defined as permanent absence of menses (at least 12 months since the last menstrual period). The body mass index (BMI) was calculated as the current self-reported body weight (kg) divided by the height squared (m^2^). The metabolic equivalent (MET) hours per week was used to estimate physical activity, and the detailed methods of calculating MET have been described previously (19).

### Assessment of dietary fatty acid intake

Dietary *n* **–** 3 PUFA intake during the previous year was collected via a validated 81-item food frequency questionnaire (FFQ) (20). For each food item, a standard portion size was specified and the frequency of consumption was questioned. Energy and fatty acids intakes were calculated based on the China Food Composition Table (21). Our study measured the following *n* **–** 3 PUFA intake variables: ALA, long-chain *n* **–** 3 PUFAs (EPA + DPA + DHA) and total *n* **–** 3 PUFAs (ALA + EPA + DPA + DHA). The major food sources of dietary EPA, DPA, and DHA are fish, seafood and fish oil. Few foods contain only one type of long-chain *n* **–** 3 PUFA. Therefore, long-chain *n* **–** 3 PUFAs were not disaggregated.

### Measurement of erythrocyte fatty acids

Fasting venous blood samples were obtained on the second day of the participants’ admission and before any medication, surgery or examination. Erythrocytes were washed three times with normal saline and separated within 2 h of collection and were stored at –80°C for subsequent analysis. Erythrocyte concentrations of fatty acids were measured by gas chromatography (GC).

The extraction of fatty acids was conducted using the method described by Folch et al. (22) with chloroform/methanol (2:1, v/v). The fatty acids were methylated with a 14% boron-trifluoride ether/methanol (1:3, v/v) solution for 60 mins at 90°C. The fatty acid methyl esters were separated using an Agilent 7890A GC system (Agilent, CA, USA) equipped with a DB-23 capillary column (60 m × 0.25 mm internal diameter × 0.15 μm film; Agilent, CA, USA) and a flame ionization detector. The analytical conditions applied were as follows: (1) nitrogen as carrier gas; (2) split ratio of 5:1 with the injection temperature at 250°C. The oven temperature started at 50°C for 1 min and was programmed from 50 to 175°C at a rate of 25°C/min, and the temperature was continuously increased to 230°C at a rate of 3.5°C/min followed by a 15-min hold period. Comparing the retention time of the samples with commercially available standards to identify individual fatty acids, the amount of each fatty acid was expressed as a percentage of the total erythrocyte membrane fatty acids (relative, %). The intra-assay coefficients of variation (CVs) and inter-assay CVs were <10 and <20% for *n* **–** 3 PUFAs.

### Statistical analysis

We used Mann-Whitney *U*-test for continuous variables and χ^2^ test for categorical variables to examine differences in characteristics between breast cancer cases and controls. Dietary *n* **–** 3 PUFA intake was energy-adjusted using the residual method (23) and then categorized as tertiles (T) on the basis of distribution among the controls. Logistic regression model was used to calculate odds ratios (ORs) and 95% confidence intervals (CIs) for breast cancer risk in relation to dietary *n* **–** 3 PUFAs. Pearson correlation coefficients were calculated between dietary *n* **–** 3 PUFAs and each erythrocyte *n* **–** 3 PUFA. We tested the mediating effect of erythrocyte *n* **–** 3 PUFAs using a PROCESS plug-in application for SPSS 25.0 provided by Preacher and Hayes (24), where the mediator should be the continuous variable. The mediation analysis model can be expressed by the following three regression equations (25). First, regressing the independent variable X on the dependent variable Y. The main effect is a precondition for the mediating effect, and the regression effect *c* must be significant (regression Equation 1). Second, regression Equation 2 explains the effect of X on the mediating variable M. When the regression coefficient *a* is significant, it indicates the existence of an effect of the independent variable on the mediating variable. Third, regressing the X and M on Y simultaneously. It reveals the association between X and Y adjusted for M and the association between M and Y adjusted for X (regression Equation 3).


(1) $$\text{Y}=i+c\,\text{X}+e_{1}$$

(2) $$\text{M}=i+a\,\text{X}+e_{2}$$

(3) Y=i+c⁢X′+b⁢M+e3

A bootstrapping method was applied to test the significance of mediating effect, which has high statistical power (26). In the serial mediation model, there was a sequential relationship between the mediating variables (27). Model 6 was chosen with a bootstrapped sample size of 5,000, with dietary *n* **–** 3 PUFAs as X, breast cancer as Y, and erythrocyte *n* **–** 3 PUFAs as M at 95% confidence interval. A significant mediation effect was established if zero was not between the lower and upper bound. We calculated the direct, indirect and total effects after adjusting for age, BMI, MET-h/week, education, passive smoking, regular drinking, first-degree relatives with cancer and energy intake.

Analyses were performed using SPSS version 25.0 (IBM Corp, Armonk, NY, USA). A two-sided *P* value < 0.05 indicated statistically significant.

## Results

### Participant characteristics

The characteristics of the cases and the control subjects are shown in Table 1. Compared with controls, cases were more likely to have higher BMI, to drink regularly, and have a family history of cancer in first-degree relatives. Cases were also more likely to have lower levels of education and household and recreational activity. Cases consumed lower ALA, long-chain *n* **–** 3 PUFAs and total *n* **–** 3 PUFAs than those of controls. Besides, erythrocyte individual and total *n* **–** 3 PUFAs proportions in cases were lower than those in controls.

**TABLE 1:** General characteristics of the study subjects^a^.

Variables	Case (*n* = 850)	Control (*n* = 861)	*P*-value
Age (years) mean ± SD	48.33 ± 9.59	48.33 ± 9.53	0.991
BMI (kg/m^2^) mean ± SD	23.32 ± 3.70	22.68 ± 3.36	<0.001
Household and recreational activities, MET-h/week (mean ± SD)	36.47 ± 23.73	40.50 ± 24.91	0.001
Occupation [*n* (%)]			0.647
Administrator/other white-collar workers	176 (20.71)	190 (22.07)	
Blue-collar worker	233 (27.41)	243 (28.22)	
Farmer/other	441 (51.88)	428 (49.71)	
Education [*n* (%)]			0.001
Primary school or below	209 (24.59)	231 (26.83)	
Secondary school	257 (30.24)	198 (22.99)	
High school	198 (23.29)	190 (22.07)	
College or above	186 (21.88)	242 (28.11)	
Income, Yuan/month [*n* (%)]			0.192
≤2,000	50 (5.88)	47 (5.46)	
2,001–5,000	257 (30.24)	223 (25.90)	
5,001–8,000	307 (36.12)	325 (37.75)	
≥8,001	236 (27.76)	266 (30.89)	
Regular smoker [*n* (%)]	12 (1.41)	10 (1.16)	0.646
Regular drinker [*n* (%)]	76 (8.94)	45 (5.23)	0.003
Passive smoker [*n* (%)]	319 (37.53)	327 (37.98)	0.848
Menopausal status [*n* (%)]			0.998
Premenopausal	537 (63.18)	544 (63.18)	
Postmenopausal	313 (36.82)	317 (36.82)	
First-degree relatives with cancer [*n* (%)]	126 (14.82)	81 (9.41)	0.001
Energy intake (kcal/day) mean ± SD	1511.09 ± 405.93	1544.99 ± 399.33	0.081
Dietary fatty acid intake (mean ± SD)^b^			
ALA (g/day)	0.75 ± 0.22	0.77 ± 0.22	0.007
Long-chain *n* **–** 3 PUFAs (mg/day)	57.04 ± 6.65	59.51 ± 6.52	0.024
Total *n* **–** 3 PUFAs (g/day)	0.81 ± 0.24	0.83 ± 0.23	0.005
Erythrocyte PUFAs, % of total fatty acids, mean ± SD			
ALA	0.41 ± 0.17	0.45 ± 0.18	<0.001
EPA	0.84 ± 0.42	0.94 ± 0.52	<0.001
DPA	1.09 ± 0.54	1.21 ± 0.66	<0.001
DHA	3.20 ± 0.80	3.40 ± 0.86	<0.001
Total *n* **–** 3 PUFAs	5.72 ± 1.52	6.35 ± 1.75	<0.001

ALA, α-linolenic acid; DHA, docosahexaenoic acid; DPA, docosapentaenoic acid; EPA, Eicosapentaenoic acid; PUFA polyunsaturated fatty acid; SD standard deviation.

^a^Mann-Whitney *U*-test was used for the comparison of continuous variables between cases and controls. Chi-square test was used to test the differences of categorical variables.

^b^Dietary intakes were adjusted for total energy using the residual method.

### Associations between dietary *n* **–** 3 polyunsaturated fatty acids and breast cancer risk

As presented in Table 2, after adjusting for potential covariates, higher intake of dietary ALA, long-chain *n* **–** 3 PUFAs and total *n* **–** 3 PUFAs was associated with decreased risk of breast cancer. The adjusted OR_*tertile3*_ *_*vs*_*_*tertile1*_ (95% CI) was 0.70 (0.55, 0.90) for ALA, 0.76 (0.60, 0.97) for long-chain *n* **–** 3 PUFAs and 0.74 (0.58, 0.94) for total *n* **–** 3 PUFAs, respectively.

**TABLE 2:** Associations between dietary *n* **–** 3 PUFAs and breast cancer risk^a^.

	Cases/Controls	Crude-OR (95% CI)	*P*-trend	Adjusted-OR (95% CI)	*P*-trend
Dietary ALA			0.005		0.005
T1	328/287	1.00 (Ref.)		1.00 (Ref.)	
T2	287/287	0.88 (0.70–1.10)		0.89 (0.70–1.12)	
T3	235/287	0.72 (0.57–0.91)		0.70 (0.55–0.90)	
Dietary long-chain *n* **–** 3 PUFAs			0.010		0.025
T1	340/287	1.00 (Ref.)		1.00 (Ref.)	
T2	255/287	0.75 (0.59–0.94)		0.77 (0.60–0.97)	
T3	255/287	0.74 (0.59–0.94)		0.76 (0.60–0.97)	
Dietary total *n* **–** 3 PUFAs			0.014		0.015
T1	319/287	1.00 (Ref.)		1.00 (Ref.)	
T2	294/287	0.92 (0.73–1.16)		0.95 (0.75–1.20)	
T3	237/287	0.74 (0.59–0.94)		0.74 (0.58–0.94)	

ALA, α-linolenic acid; CI, confidence interval; OR, odds ratio; PUFA, polyunsaturated fatty acid; T, tertile.

^a^Adjusted for age, BMI, MET-h/week, education, passive smoking, regular drinking, first-degree relatives with cancer and energy intake.

### Correlation analyses

In control subjects, erythrocyte ALA was not related to any dietary *n* **–** 3 PUFA intake (see Table 3, all *P* > 0.05). Positive correlations were observed between dietary long-chain *n* **–** 3 PUFAs and erythrocyte long-chain *n* **–** 3 PUFAs (*r* = 0.029 for EPA, 0.085 for DPA and 0.147 for DHA, all *P* < 0.05). Meanwhile, higher intake of total *n* **–** 3 PUFAs was related to higher proportions of erythrocyte EPA and DHA (*r* = 0.115 and 0.078, respectively, all *P* < 0.05), but not DPA (*r* = 0.048, *P* > 0.05). Erythrocyte *n* **–** 3 PUFAs were significantly and positively correlated with each other (*r* range from 0.082 to 0.445, all *P* < 0.05).

**TABLE 3:** Correlations between dietary and erythrocyte *n* **–** 3 PUFAs in control subjects.

	(1) Dietary ALA	(2) Dietary long-chain *n* – 3 PUFAs	(3) Dietary total *n* – 3 PUFAs	(4) Erythrocyte ALA	(5) Erythrocyte EPA	(6) Erythrocyte DPA	(7) Erythrocyte DHA
(1) Dietary ALA	1.000						
(2) Dietary long-chain *n* **–** 3 PUFAs	–	1.000					
(3) Dietary total *n* **–** 3 PUFAs	–	–	1.000				
(4) Erythrocyte ALA	–0.030	0.031	–0.019	1.000			
(5) Erythrocyte EPA	0.056	0.029**	0.115**	0.142**	1.000		
(6) Erythrocyte DPA	0.019	0.085*	0.048	0.154**	0.445**	1.000	
(7) Erythrocyte DHA	0.047	0.147**	0.078*	0.082*	0.171**	0.120**	1.000

ALA, a-linolenic acid; DHA, docosahexaenoic acid; DPA, docosapentaenoic acid; EPA, eicosapentaenoic acid; PUFA, polyunsaturated fatty acid.

**P <0.01, *P <0.05.

### Mediation analysis on dietary α-linolenic acid and breast cancer risk

**FIGURE 1:**
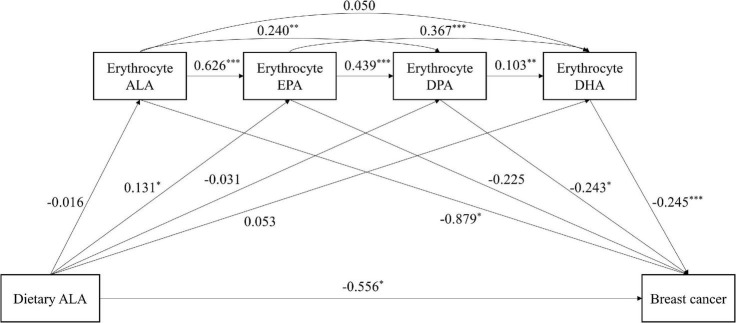
The chain mediating effect of erythrocyte *n* **–** 3 PUFAs on the relationship between dietary ALA intake and breast cancer risk. ALA, α-linolenic acid; DHA, docosahexaenoic acid; DPA, docosapentaenoic acid; EPA, eicosapentaenoic acid; PUFA, polyunsaturated fatty acid. Standardized estimating of 5,000 bootstrap sample. Age, BMI, MET-h/week, education, passive smoking, regular drinking, first-degree relatives with cancer and energy intake were controlled. ^***^P < 0.001, ^**^P < 0.01, *P < 0.05.

The results of the mediation analysis on dietary ALA intake and breast cancer risk are reported in Table 4 and Figure 1. The bootstrap analyses revealed three significant indirect effects between dietary ALA intake and breast cancer risk. The direct effect was significant because zero falls outside the confidence intervals [direct effect (95% CI) = –0.556 (–1.018, –0.093)]. The indirect effect eight and nine indicated that higher intake of dietary ALA was associated with higher levels of erythrocyte EPA, which in turn led to higher levels of DPA or DHA and finally associated with the risk of breast cancer [indirect effect (95% CI) = –0.014 (–0.033, –0.002) and –0.012 (–0.026, –0.003), respectively]. Furthermore, in the multiple chain mediation test of erythrocyte EPA, DPA and DHA, the 95% CI of indirect effect fourteen does not contain 0, and the mediation effect is significant [(indirect effect (95% CI) = –0.001 (–0.004, –0.0002)] However, the total indirect effect was not significant from dietary ALA intake to breast cancer risk through erythrocyte *n* **–** 3 PUFAs [total indirect effect (95% CI) = –0.042 (–0.144, 0.049)].

**TABLE 4:** Direct and indirect effect of the chain mediation model with mediator of erythrocyte *n* **–** 3 PUFAs in association between dietary ALA intake and breast cancer risk^a^.

	Effect	95% CI	SE
**Direct effect**			
Dietary ALA → breast cancer	–0.556	-1.018, -0.093	0.236
**Indirect effect**			
(1) Dietary ALA → RALA → breast cancer	0.014	–0.024, 0.057	0.020
(2) Dietary ALA → REPA → breast cancer	–0.029	–0.082, 0.003	0.022
(3) Dietary ALA → RDPA → breast cancer	0.008	–0.031, 0.047	0.019
(4) Dietary ALA → RDHA → breast cancer	–0.013	–0.062, 0.030	0.023
(5) Dietary ALA → RALA → REPA → breast cancer	0.002	–0.004, 0.011	0.004
(6) Dietary ALA → RALA → RDPA → breast cancer	0.001	–0.002, 0.005	0.002
(7) Dietary ALA → RALA → RDHA → breast cancer	0.0002	–0.001, 0.002	0.001
(8) Dietary ALA → REPA → RDPA → breast cancer	–0.014	–0.033, –0.002	0.008
(9) Dietary ALA → REPA → RDHA → breast cancer	–0.012	–0.026, –0.003	0.006
(10) Dietary ALA → RDPA → RDHA → breast cancer	0.001	–0.002, 0.007	0.002
(11) Dietary ALA → RALA → REPA → RDPA → breast cancer	0.001	–0.002, 0.005	0.002
(12) Dietary ALA → RALA → REPA → RDHA → breast cancer	0.001	–0.002, 0.004	0.001
(13) Dietary ALA → RALA → RDPA → RDHA → breast cancer	0.0001	–0.0002, 0.0005	0.0002
(14) Dietary ALA → REPA → RDPA → RDHA → breast cancer	–0.001	–0.004, –0.0002	0.001
(15) Dietary ALA → RALA → REPA → RDPA → RDHA → breast cancer	0.0001	–0.0002, 0.0005	0.0002
**Total indirect effect**			
Dietary ALA → breast cancer	–0.042	-0.144, 0.049	0.049

RALA, erythrocyte α-linolenic acid; CI, confidence interval; RDHA, erythrocyte docosahexaenoic acid; RDPA, erythrocyte docosapentaenoic acid; REPA, erythrocyte eicosapentaenoic acid; PUFA, polyunsaturated fatty acid; SE, standard error.

^a^Adjusted for age, BMI, MET-h/week, education, passive smoking, regular drinking, first-degree relatives with cancer and energy intake.

### Mediation analysis on dietary long-chain *n* **–** 3 polyunsaturated fatty acids and breast cancer risk

**FIGURE 2:**
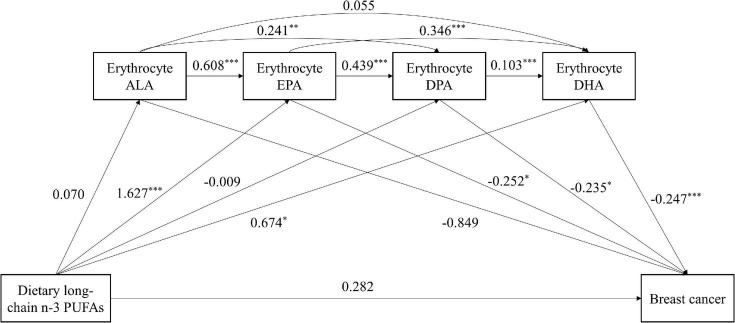
The chain mediating effect of erythrocyte *n* **–** 3 PUFAs on the relationship between dietary long-chain *n* **–** 3 PUFA intake and breast cancer risk. ALA, α-linolenic acid; DHA, docosahexaenoic acid; DPA, docosapentaenoic acid; EPA, eicosapentaenoic acid; PUFA, polyunsaturated fatty acid. Standardized estimating of 5,000 bootstrap sample. Age, BMI, MET-h/week, education, passive smoking, regular drinking, first-degree relatives with cancer and energy intake were controlled. ^***^P < 0.001, ^**^P < 0.01, *P < 0.05.

Mediating analysis revealed the mediation effect on relationship between consumption of dietary long-chain *n* **–** 3 PUFAs and breast cancer risk (see Table 5 and Figure 2). The direct effect was not significant [direct effect (95% CI) = 0.282 (–1.261, 1.825)], whereas the total indirect effects was significant [total indirect effect (95% CI) = –0.982 (–1.529, –0.508)]. Erythrocyte EPA or DHA had significant indirect effect on the correlation between dietary long-chain *n* **–** 3 PUFAs and breast cancer risk [indirect effect (95% CI) = –0.409 (–0.853, –0.018) and –0.167 (–0.361, –0.025), respectively]. The chain mediation of erythrocyte EPA and DPA or DHA between dietary long-chain *n* **–** 3 PUFAs and breast cancer was significant [indirect effect (95% CI) = –0.167 (–0.332, –0.033) and –0.139 (–0.237, –0.063), respectively]. The serial mediating effect of erythrocyte EPA, DPA, and DHA was significant [indirect effect (95% CI) = –0.018 (–0.041, –0.005)]. Combined with Figure 2, erythrocyte EPA or DHA alone had a significant mediating effect. However, erythrocyte DPA can play a chain mediating role and were inversely associated with breast cancer risk only when it combined with EPA and DHA.

**TABLE 5:** Direct and indirect effect of the chain mediation model with mediator of erythrocyte *n* **–** 3 PUFAs in association between dietary long-chain *n* **–** 3 PUFA intake and breast cancer risk^a^.

	Effect	95% CI	SE
**Direct effect**			
Dietary long-chain *n* **–** 3 PUFAs → breast cancer	0.282	–1.261, 1.825	0.787
**Indirect effect**			
(1) Dietary long-chain *n* **–** 3 PUFAs → RALA → breast cancer	–0.060	–0.237, 0.070	0.075
(2) Dietary long-chain *n* **–** 3 PUFAs → REPA → breast cancer	–0.409	–0.853, –0.018	0.212
(3) Dietary long-chain *n* **–** 3 PUFAs → RDPA → breast cancer	0.002	–0.126, 0.143	0.064
(4) Dietary long-chain *n* **–** 3 PUFAs → RDHA → breast cancer	–0.167	–0.361, –0.025	0.086
(5) Dietary long-chain *n* **–** 3 PUFAs → RALA → REPA → breast cancer	–0.011	–0.049, 0.011	0.015
(6) Dietary long-chain *n* **–** 3 PUFAs → RALA → RDPA → breast cancer	–0.004	–0.018, 0.005	0.006
(7) Dietary long-chain *n* **–** 3 PUFAs → RALA → RDHA → breast cancer	–0.001	–0.009, 0.005	0.003
(8) Dietary long-chain *n* **–** 3 PUFAs → REPA → RDPA → breast cancer	–0.167	–0.332, –0.033	0.076
(9) Dietary long-chain *n* **–** 3 PUFAs → REPA → RDHA → breast cancer	–0.139	–0.237, –0.063	0.044
(10) Dietary long-chain *n* **–** 3 PUFAs → RDPA → RDHA → breast cancer	–0.0002	–0.015, 0.015	0.007
(11) Dietary long-chain *n* **–** 3 PUFAs → RALA → REPA → RDPA → breast cancer	–0.004	–0.018, 0.005	0.006
(12) Dietary long-chain *n* **–** 3 PUFAs → RALA → REPA → RDHA → breast cancer	–0.004	–0.014, 0.004	0.005
(13) Dietary long-chain *n* **–** 3 PUFAs → RALA → RDPA → RDHA → breast cancer	–0.0004	–0.002, 0.001	0.001
(14) Dietary long-chain *n* **–** 3 PUFAs → REPA → RDPA → RDHA → breast cancer	–0.018	–0.041, –0.005	0.009
(15) Dietary long-chain *n* **–** 3 PUFAs → RALA → REPA → RDPA → RDHA → breast cancer	–0.001	–0.002, 0.001	0.001
**Total indirect effect**			
Dietary long-chain *n* **–** 3 PUFAs → breast cancer	–0.982	–1.529, –0.508	0.261

RALA, erythrocyte α-linolenic acid; CI, confidence interval; RDHA, erythrocyte docosahexaenoic acid; RDPA, erythrocyte docosapentaenoic acid; REPA, erythrocyte eicosapentaenoic acid; PUFA, polyunsaturated fatty acid; SE, standard error.

^a^Adjusted for age, BMI, MET-h/week, education, passive smoking, regular drinking, first-degree relatives with cancer and energy intake.

### Mediation analysis on dietary total *n* **–** 3 polyunsaturated fatty acids and breast cancer risk

**FIGURE 3:**
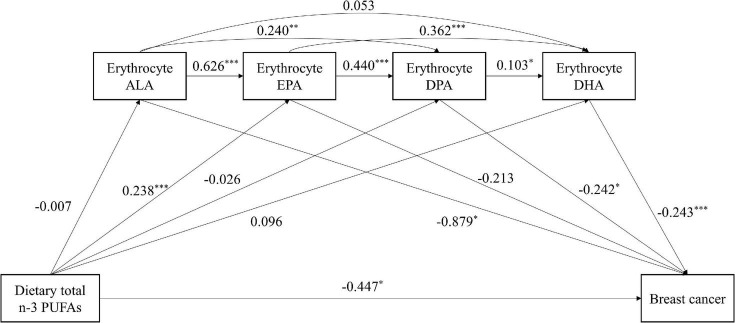
The chain mediating effect of erythrocyte *n* **–** 3 PUFAs on the relationship between dietary total *n* **–** 3 PUFA intake and breast cancer risk. ALA, α-linolenic acid; DHA, docosahexaenoic acid; DPA, docosapentaenoic acid; EPA, eicosapentaenoic acid; PUFA, polyunsaturated fatty acid. Standardized estimating of 5,000 bootstrap sample. Age, BMI, MET-h/week, education, passive smoking, regular drinking, first-degree relatives with cancer and energy intake were controlled. ^***^P < 0.001, ^**^P < 0.01, *P < 0.05.

Table 6 and Figure 3 outlined the mediating effect of mediators in association between dietary total *n* **–** 3 PUFAs and breast cancer risk. Significant direct effect and total indirect effect were found [direct effect (95% CI) = –0.447 (–0.873, –0.022); total indirect effect (95% CI) = –0.107 (–0.216, –0.014)]. The total mediating effect of dietary total *n* **–** 3 PUFAs on risk of breast cancer was 19.31%. Erythrocyte EPA and DPA partially mediated the effect of dietary total *n* **–** 3 PUFAs and breast cancer risk [indirect effect (95% CI) = –0.025 (–0.053, –0.006)]. Erythrocyte EPA and DPA accounted for 4.51% on the correlation between dietary total *n* **–** 3 PUFAs and breast cancer risk. Similarly, erythrocyte EPA and DHA partially mediated the effect [indirect effect (95% CI) = –0.021 (–0.039, –0.008)], accounting for a mediation ratio of 3.79%. The association between dietary total *n* **–** 3 PUFA intake and breast cancer risk was partly mediated by erythrocyte EPA, DPA, and DHA sequentially [indirect effect (95% CI) = –0.003 (–0.006, –0.001)], accounting for a mediation ratio of 0.54%.

**TABLE 6:** Direct and indirect effect of the chain mediation model with mediator of erythrocyte *n* **–** 3 PUFAs in association between dietary total *n* **–** 3 PUFA intake and breast cancer risk^a^.

	Effect	95% CI	SE	Proportion mediated (%)
**Direct effect**				
Dietary total *n* **–** 3 PUFAs → breast cancer	–0.447	–0.873, –0.022	0.217	
**Indirect effect**				
(1) Dietary total *n* **–** 3 PUFAs → RALA → breast cancer	0.007	–0.032, 0.044	0.018	N/A
(2) Dietary total *n* **–** 3 PUFAs → REPA → breast cancer	–0.051	–0.122, 0.007	0.032	N/A
(3) Dietary total *n* **–** 3 PUFAs → RDPA → breast cancer	0.006	–0.029, 0.043	0.018	N/A
(4) Dietary total *n* **–** 3 PUFAs → RDHA → breast cancer	–0.023	–0.072, 0.014	0.021	N/A
(5) Dietary total *n* **–** 3 PUFAs → RALA → REPA → breast cancer	0.001	–0.005, 0.008	0.003	N/A
(6) Dietary total *n* **–** 3 PUFAs → RALA → RDPA → breast cancer	0.0004	–0.002, 0.004	0.001	N/A
(7) Dietary total *n* **–** 3 PUFAs → RALA → RDHA → breast cancer	0.0001	–0.001, 0.002	0.001	N/A
(8) Dietary total *n* **–** 3 PUFAs → REPA → RDPA → breast cancer	–0.025	–0.053, –0.006	0.012	4.51
(9) Dietary total *n* **–** 3 PUFAs → REPA → RDHA → breast cancer	–0.021	–0.039, –0.008	0.008	3.79
(10) Dietary total *n* **–** 3 PUFAs → RDPA → RDHA → breast cancer	0.001	–0.002, 0.006	0.002	N/A
(11) Dietary total *n* **–** 3 PUFAs → RALA → REPA → RDPA → breast cancer	0.001	–0.002, 0.004	0.001	N/A
(12) Dietary total *n* **–** 3 PUFAs → RALA → REPA → RDHA → breast cancer	0.0004	–0.002, 0.003	0.001	N/A
(13) Dietary total *n* **–** 3 PUFAs → RALA → RDPA → RDHA → breast cancer	0.0000	–0.0002, 0.0004	0.0001	N/A
(14) Dietary total *n* **–** 3 PUFAs → REPA → RDPA → RDHA → breast cancer	–0.003	–0.006, –0.001	0.001	0.54
(15) Dietary total *n* **–** 3 PUFAs → RALA → REPA → RDPA → RDHA → breast cancer	0.0001	–0.0003, 0.0004	0.0002	N/A
**Total indirect effect**				
Dietary total *n* **–** 3 PUFAs → breast cancer	–0.107	–0.216, –0.014	0.051	19.31

RALA, erythrocyte α-linolenic acid; CI, confidence interval; RDHA, erythrocyte docosahexaenoic acid; RDPA, erythrocyte docosapentaenoic acid; REPA, erythrocyte eicosapentaenoic acid; PUFA, polyunsaturated fatty acid; SE, standard error.

^a^Adjusted for age, BMI, MET-h/week, education, passive smoking, regular drinking, first-degree relatives with cancer and energy intake.

^b^N/A is due to no mediating effect.

## Discussion

The current study examined the association between dietary intake of *n* **–** 3 PUFAs and breast cancer risk, and further explored the serial mediating role of erythrocyte *n* **–** 3 PUFAs within the linkage of dietary *n* **–** 3 PUFAs and breast cancer risk. The inverse association of dietary long-chain or total *n* **–** 3 PUFAs with breast cancer risk could be accounted for the potential beneficial actions of dietary long-chain or total *n* **–** 3 PUFAs on increasing erythrocyte long chain *n* **–** 3 PUFAs. However, erythrocyte *n* **–** 3 PUFAs were not mediators in the association between dietary ALA and breast cancer risk.

The present study showed that the total indirect effect of erythrocyte *n* **–** 3 PUFAs was not significant in the association between dietary ALA intake and breast cancer risk. There are some possible explanations. First, we failed to find the indirect effect of erythrocyte ALA on the relationship between dietary ALA and breast cancer. ALA, an essential fatty acid, cannot be produced endogenously and is closely tied to dietary exposure. However, consistent with previous studies (28, 29), dietary ALA was not found to be associated with erythrocyte ALA in the present study. ALA was oxidized by families of enzymes, including cyclooxygenases, lipoxygenases and cytochrome P450 enzymes (30). ALA converted to oxylipins at a higher rate and in a higher amount, which might be the reason why ALA did not accumulate in tissue as much as long-chain *n* **–** 3 PUFAs (31). Moreover, a competition between ALA and linoleic acid (LA) may interfere with the correlation between dietary and circulating ALA. Only high supplementation of dietary ALA was reported to result in modest increases in plasma ALA concentrations, because the LA concurrently intake could reduce ALA accumulation (32). Second, we observed a direct effect of dietary ALA on breast cancer risk, but not mediated by erythrocyte ALA. ALA originated from a variety of foods, such as vegetable oils, green leafy vegetables, and common seeds and nuts (5). Consumption of other nutrients from ALA-rich foods might be important confounding factors underlying the inverse relationship between dietary ALA and breast cancer risk. Third, we observed that the association between dietary ALA and breast cancer risk could be partially explained by erythrocyte long-chain *n* **–** 3 PUFAs. This might be that long-chain *n* **–** 3 PUFAs can also be metabolized from ALA in *vivo* by successive desaturation and elongation reactions (33), although the conversion efficiency was poor. The conversion rate of ALA to EPA is 21% in women with a higher activity of delta-6 desaturase expression caused by sex hormones (34), while the conversions rate of ALA to DPA or DHA appear to be lower (6 and 9%, respectively) (35, 36). All of these might explain why the total indirect effect of erythrocyte *n* **–** 3 PUFAs on breast cancer risk was not significant.

The inverse association between dietary long-chain *n* **–** 3 PUFAs and breast cancer risk was fully mediated by erythrocyte long-chain *n* **–** 3 PUFAs. Our study showed that the consumption of long-chain *n* **–** 3 PUFAs was significantly associated with erythrocyte EPA, DPA and DHA. Consistent with our results, other researches also reported that dietary EPA and DHA was positively associated with the levels of erythrocyte EPA and DHA (37, 38). Previous studies observed inverse associations between erythrocyte long-chain *n* **–** 3 PUFAs and breast cancer risk (39, 40). As a result, the association between dietary long-chain *n* **–** 3 PUFAs might be mediated by erythrocyte long-chain *n* **–** 3 PUFAs. Notably, the omega-3 index is defined as the percentage of erythrocyte EPA plus DHA in total fatty acids and was thought to be associated with lower risk for health events, especially cardiovascular events (41). We also demonstrated the relationship between erythrocyte EPA and DHA and dietary long-chain *n* **–** 3 PUFAs and their mediating roles in the association between dietary long-chain *n* **–** 3 PUFAs/total *n* **–** 3 PUFAs and breast cancer. Therefore, it is worthwhile for future research to explore the relationship between omega-3 index and health events such as cancer. In terms of our observation of the fully mediated effect of erythrocyte long-chain *n* **–** 3 PUFAs, a few more points needed to be interpreted with care. It should be noted that the full mediation depends on whether the regression coefficient *c’* is significant, and the significance of *c’* is affected by the sample size. When a large enough sample is collected, the previous conclusion of full mediation may become partial mediation. Furthermore, full mediation actually still implies that there may be other mediating variables (42).

The data herein demonstrated that erythrocyte DPA not independently, but sequentially mediated (with EPA and DHA) the relationship between dietary long-chain *n* **–** 3 PUFAs and breast cancer risk. Dyall et al. (43) reviewed studies on metabolic differences among long-chain *n* **–** 3 PUFAs and noted the potential role of DPA. It might serve as a reservoir for EPA and DHA. Several reports indicate that interconversion of EPA, DPA, and DHA via retro-conversion and elongation pathways may occur (44, 45). DPA supplementation could increase the plasma concentrations of several arachidonic acid (AA)-derivative, including total PGE_2_ levels and other AA- and dihomo-gamma-linolenic acid-derived PG species (44). AA-derived metabolites contribute to angiogenesis (46). The DPA alone therefore could not play a mediating role in the inverse association between dietary long-chain *n* **–** 3 PUFAs and breast cancer risk, but rather plays a mediating role through interconversion with EPA and DHA. We also observed that the indirect effect of erythrocyte EPA was higher than erythrocyte DHA. It may be due to the difference in metabolism and incorporation of EPA and DHA in different blood components. Brown et al. (47) demonstrated that erythrocyte EPA was a stronger indicator of *n* **–** 3 PUFA intake than DHA. DHA is incorporated into the inner erythrocyte leaflet and are more influenced by erythrocyte turnover, whereas EPA is incorporated into the outer erythrocyte leaflet and its content largely depends on the equilibrium with plasma (47).

We found that the association between total consumed *n* **–** 3 PUFAs and breast cancer risk was partly mediated by erythrocyte long-chain *n* **–** 3 PUFAs. As mentioned above, the source of dietary ALA was complex and diverse. For example, the inverse association of ALA from fruits and vegetables with the risk of breast cancer might be associated with other compounds in fruits and vegetables, such as folate (48) or fiber (49). Given the fact that ALA accounts for the majority of dietary total *n* **–** 3 PUFAs [the proportion of dietary ALA to total dietary *n* **–** 3 PUFAs in cases and controls was 93.17 (±6.42)% and 93.32 (±6.41)%, respectively], it was possible that the relationship between total *n* **–** 3 PUFAs and breast cancer risk was influenced by other nutrients. The partial mediation implied that there might be other mediators’ worth exploring in the association between dietary total *n* **–** 3 PUFAs and breast cancer risk. For example, it was found that mice fed with diets containing increasing amounts of EPA + DHA had decreasing levels of erythrocyte *n* **-** 6 PUFAs and increasing levels of erythrocyte saturated fatty acids (SFAs) (50). Prisco et al. illustrated that healthy male volunteers supplemented with *n* **–** 3 PUFAs for 4 months had decreased level of erythrocyte *n*-6 PUFAs and increased level of erythrocyte monounsaturated fatty acids (MUFAs) (51). This indicated that erythrocyte SFAs, MUFAs, and *n*-6 PUFAs may also be mediators of the association dietary *n* **–** 3 PUFAs and breast cancer risk.

Strengths of this study include the relatively large sample size and a number of potential confounders. We were further able to apply mediation analysis of four mediators on the association between dietary *n* **–** 3 PUFAs consumed and breast cancer risk for the first time. Several limitations should be taken into account. Firstly, as a case-control study, the causal sequence of variables cannot be curtained. Longitudinal studies were needed to verify the causality of all variables in the future. Secondly, subjective self-report and recall bias might exist. To diminish it, we used photographs with usual portion size of foods to obtain intake as accurately as possible and recruited patients who were diagnosed less than 3 months before the interview. Thirdly, we only had a single measure of erythrocyte *n* **–** 3 PUFAs, which may not ideally reflect long-term exposure. However, one study showed that no statistically significant increase was observed in the level of *n* **–** 3 PUFAs over time in the Cardiovascular Health Study across 13 consecutive years of measures (52). Finally, we cannot completely deny that there may still be some residual confounding factors and these results should be extended to the general population in other regions for confirmation.

## Conclusion

Our findings suggest that the influence of dietary total *n* **–** 3 PUFAs on breast cancer risk was at least partially explained by beneficial effects of total *n* **–** 3 PUFA intake on increasing the erythrocyte long-chain *n* **–** 3 PUFAs. Importantly, erythrocyte long-chain *n* **–** 3 PUFAs may fully explain the protective effect of consumed long-chain *n* **–** 3 PUFAs on breast cancer risk. The links between dietary *n* **–** 3 PUFAs and breast cancer risk may be mediated by erythrocyte long-chain *n* **–** 3 PUFAs, whereas evidence of a mediating role of erythrocyte ALA was not supported. This study highlights the complexity in using a simple analysis of dietary individual *n* **–** 3 PUFA to predict breast cancer risk without considering the variety of metabolic processes. We demonstrated the possibility of erythrocyte *n* **–** 3 PUFAs to interpret the role of dietary *n* **–** 3 PUFAs in breast cancer risk. Interventions aimed at increasing erythrocyte long-chain *n* **–** 3 PUFAs may represent a promising strategy for breast cancer prevention. Identifying the ratios between long-chain *n* **–** 3 PUFAs that are best for human health, including those between *n*-6 PUFAs could be investigated in future studies.

## Data availability statement

The raw data supporting the conclusions of this article will be made available by the authors, without undue reservation.

## Ethics statement

The studies involving human participants were reviewed and approved by the Ethical Committee of the School of Public Health, Sun Yat-sen University. The patients/participants provided their written informed consent to participate in this study.

## Author contributions

ZZ collected the data, performed the experiments, analyzed the data, and wrote the manuscript. YJ, XL, DS, TM, and RZ participated in data collection and experiments. CZ was responsible for designing and writing grants, supervision of the research, and wrote the manuscript. All authors contributed to the article and approved the submitted version.
